# Potential Synergy between Spores of *Metarhizium anisopliae* and Plant Secondary Metabolite, 1-Chlorooctadecane for Effective Natural Acaricide Development

**DOI:** 10.3390/molecules25081900

**Published:** 2020-04-20

**Authors:** Abid Hussain, Ahmed Mohammed AlJabr

**Affiliations:** 1 Laboratory of Bio-Control and Molecular Biology, Department of Arid Land Agriculture, College of Agricultural and Food Sciences, King Faisal University, Hofuf 31982, Al-Ahsa, Saudi Arabia; solvia_aah@yahoo.com; 2 Research and Consulting Institute, King Faisal University, Hofuf 31982, Al-Ahsa, Saudi Arabia; 3 Ministry of Environment, Water and Agriculture, Riyadh 11442, Saudi Arabia

**Keywords:** antioxidants, compatibility, date palm dust mites, host defense, *Oligonychus afrasiaticus*, *Metarhizium anisopliae*, natural acaricide, synergism, 1-Chlorooctadecane

## Abstract

Date palm dust mites are important pests severely infesting valuable nutritious fruits (dates) of date palm. In search of an alternative to acaricides, joint action of *Metarhizium anisopliae* EBCL 02049 spores and 1-Chlorooctadecane was evaluated as a potential candidate for the management of *Oligonychus afrasiaticus* through natural products. In this regard, in vitro tests were performed to evaluate the interaction of *M. anisopliae* spores with multiple doses of 1-Chlorooctadecane (0.8, 1.6, 2.4, 3.2, and 4.0 mg/mL). Compatibility bioassay results evidenced from vegetative growth (77.7–84.40 mm), sporulation (5.50–7.30 × 10^6^ spores/mL), and germination (96.70–98.20%), revealed that all the tested doses are compatible (biological index > 82) with the spores of *M. anisopliae*. The impact of combined treatment of spores with 1-Chlorooctadecane in different proportions (Scheme I, II, III, and IV) compared to their sole application against *O. afrasiaticus* was evaluated by concentration–mortality response bioassays. Results showed that all the combined treatments revealed high mortality compared to the sole application, which showed relatively slow mortality response over time. Toxicity recorded from Scheme IV combinations (80% 1-Chlorooctadecane: 20% Spores), exhibited strong synergistic interaction (joint toxicity = 713). Furthermore, potent interactions have overcome the host antioxidant defense at the final stage of infection by tremendously reducing catalase, and superoxide dismutase activities. These experiments demonstrated fungal–toxin joint synergistic interaction as a promising date palm dust mite management option.

## 1. Introduction

Date palm (*Phoenix dactylifera* L.) is one of the oldest domesticated fruit trees with unique nutritional characteristics and great socioeconomic importance for the Middle East and North African countries. This cash crop, in addition to commercial and nutritional values, can successfully be grown in the deserts because of minimum water requirement and highly tolerance to salinity and harsh weather conditions [1]. In the Kingdom of Saudi Arabia, date palms are grown on vast areas of land, accounting for about 25% of the World’s date palms (30 million). According to an estimate, approximately 450 different cultivars out of 2000 are grown here in Saudi Arabia [2].

The economic importance of date palms is mainly because of valuable nutritious fruit (dates), which contains carbohydrates, dietary fibers, fat, lipids, minerals and protein [3]. Saudi Arabia stands second (1,302,859 tons), after the major producer Egypt (1,562,171 tons), in the world in the production of dates [4]. However, the annual yield of dates in Saudi Arabia compared to the other countries is still low might because of pest infestations. Date palm dust mites, *O. afrasiaticus* (Acari: Tetranychidae) is considered a major pest of date palms in Saudi Arabia. The weather conditions prevailing in the Kingdom and neighboring countries including Qatar, Sultanate of Oman, United Arab Emirates, Yemen, Libya, Iraq, and Egypt greatly favor *O. afrasiaticus* growth and development.

The infestation of *O. afrasiaticus* mainly depends on the moisture contents of the dates. The attack of mites starts at the *Kimri* stage (greenish), during this stage these greenish dates tremendously increase in sugar contents and moisture level that ultimately led to an increase in size and weight. At this stage, mites start spinning the web around the bunches of dates. Dust particles and exuviae from different stages are trapped into these webs resulting in the dusty appearance of bunches. Under these conditions, mites grow rapidly and multiply logarithmically [5].

Currently, management of date palm dust mites heavily relied on the use of acaricides. The use of synthetic pesticides to manage mites on dates may cause serious health hazards because the fruit contains pesticide residues. Furthermore, the use of pesticides is discouraged because of (1) environmental pollution, (2) applicator safety issues, (3) detrimental effects on non-target animals and (4) decrease in biodiversity. These shortcomings prompted the policymakers and researchers to focus on the use of alternative environmentally friendly management strategies to manage target pest species. Among alternative methods, plant-based pesticides and naturally occurring bio-control agents attained considerable importance because of their high degree of host specificity and high searching ability, respectively. The use of biopesticides to manage other mite species have been described in numerous investigations [6,7,8,9,10,11,12]. The spores of the strain of *M. anisopliae* selected for the current study had shown virulence against various pest species including *Ocinara varians* Walker [13], *Coptotermes formosanus* Shiraki [14,15], and *O. afrasiaticus* (McGregor) [16]. On the other hand, plant secondary metabolite, 1-Chlorooctadecane selected for the current study is a promising pest management candidate known to extract from numerous plant species including *Albertisia papuana* Becc [17], *Syzygium cumini* (L.) [18], *Arisaema amurense* Maxim [19], and fungus, *Trichoderma harzianum* [20]. 1-Chlorooctadecane (CH_3_ (CH_2_) 16CH_2_ Cl) was screened from our preliminary study on the chemical fractionation of *Cucumis sativus* that showed toxicity against date palm dust mites. The successful implementation of these agents can yield numerous benefits such as no side effects, cost-effectiveness, reduced reliance on conventional pesticides, minimum environmental disturbance and self-perpetuation. However, few studies have explored the potential of biopesticides to manage *O. afrasiaticus* populations [16,21,22,23]. The current study is primarily aimed to fully exploit for the first time the biocontrol potential of plant secondary metabolite, 1-Chlorooctadecane and pathogenic fungus, *M. anisopliae* EBCL 02049 by a series of experimentation including compatibility assays, concentration–mortality response bioassays, physiological enzymatic regulations, and joint toxicity index analysis in order to facilitate a knowledge-based eco-friendly date palm dust mites management approach.

## 2. Results

### 2.1. Compatibility Bioassays

The spores of *M. anisopliae* EBCL 02049 were found to be compatible because they showed a very high value of biological index (BI > 82), against all the tested concentrations of 1-Chlorooctadecane. However, we found concentration-dependent inversely proportional relationship between concentrations of 1-Chlorooctadecane and BI. All the tested concentrations of 1-Chlorooctadecane revealed significant differences in the vegetative growth (*F* = 3.32; *df* = 5, 54; *p* = 0.011), and sporulation (*F* = 3.00; *df* = 5, 54; *p* = 0.019) of *M. anisopliae* EBCL 02049. We recorded a similar concentration-dependent response. The lower concentrations failed to inhibit the fungal growth resulting in significantly wider vegetative mycelial growth and high sporulation of *M. anisopliae* EBCL 02049 (Table 1). On the other hand, spores germination of *M. anisopliae* EBCL 02049 against all the tested concentrations revealed non-significant interaction (*F* = 0.93; *df* = 5, 54; *p* = 0.468), and resulting very high percent germination (> 96%).

### 2.2. Screening Biossays

**Figure 1:**
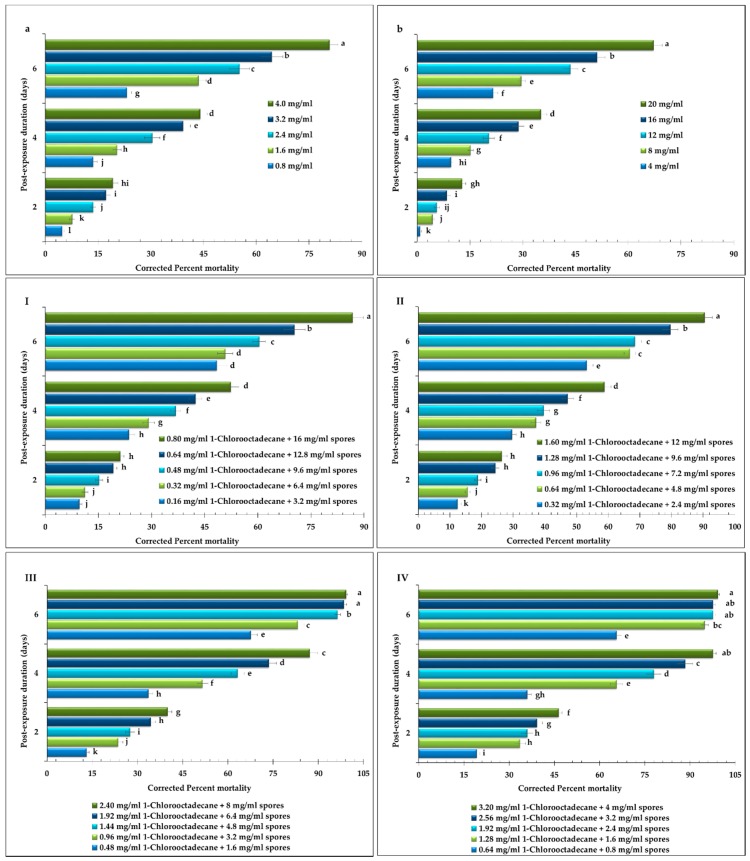
Impact of various concentrations of (**a**) 1-Chlorooctadecane; (**b**) suspensions of *M. anisopliae*; and their various interactions (**I**) 20% 1-Chlorooctadecane: 80% Spores; (**II**) 40% 1-Chlorooctadecane: 80% Spores; (**III**) 60% 1-Chlorooctadecane: 40% Spores; (**IV**) 80% 1-Chlorooctadecane: 20% Spores; on the mortality of second nymphal stage date palm dust mites. The comparisons of mortalities of *O. afrasiaticus* among different treatments were evaluated by repeated measures ANOVA (Fisher’s LSD test; α = 0.05).

Date palm dust mites exposed with different concentrations of 1-Chlorooctadecane, and *M. anisopliae* EBCL 02049 spores alone or in different proportions revealed concentration-dependent mortality response (Figure 1). The sole application of *M. anisopliae* EBCL 02049 spore suspensions at different concentrations exhibited the lowest mortality response resulting the greater value for LC_50_ (21.66 mg/mL). However, all the tested concentrations of the spores (*F* = 920.10; *df* = 4, 48; *p* < 0.0001), at different time intervals (*F* = 194.27; *df* = 2, 48; < 0.0001), and their interaction (*F* = 121.91; *df* = 8, 48; < 0.0001), revealed significant differences in the mortality of date palm dust mites (Figure 1b). On the other hand, sole application of 1-Chlorooctadecane has shown comparatively lower value for LC_50_ (3.42 mg/mL). The mortality response of date palm dust mites recorded at different time intervals (*F* = 138.93; *df* = 2, 48; < 0.0001), after different concentrations of 1-Chlorooctadecane (*F* = 1181.20; *df* = 4, 48; *p* < 0.0001), and their interaction (*F* = 140.63; *df* = 8, 48; < 0.0001) exhibited significant differences (Figure 1a).

Different bioassay schemes designed to screen the most potent interaction revealed variable interaction response in terms of the mortality of second nymphal stage of date palm dust mites. The combination of 1-Chlorooctadecane with spores mentioned in Table 2 as Scheme I (20% 1-Chlorooctadecane: 80% Spores) led to an antagonistic interaction by revealing the least joint toxicity (joint toxicity = 47). However, mortality response of date palm dust mites exposed to different concentrations of these combinations (*F* = 819.31; *df* = 4, 48; *p* < 0.0001), recorded at different time intervals (*F* = 179.31; *df* = 2, 48; < 0.0001), and their interaction (*F* = 75.94; *df* = 8, 48; < 0.0001) revealed significant differences (Figure 1I). However, the combination Scheme II in which different concentrations were prepared to finalize the final concentrations with combination strength of 40% 1-Chlorooctadecane: 60% Spores led to a synergistic interaction (joint toxicity = 112). The mortality responses of date palm dust mites displayed in the Figure 1II revealed significant differences at different concentrations (*F* = 439.53; *df* = 4, 48; *p* < 0.0001), time intervals (*F* = 265.19; *df* = 2, 48; < 0.0001), and their interaction (*F* = 26.81; *df* = 8, 48; < 0.0001).

The combined application of 1-Chlorooctadecane and spores (Scheme III: 60% 1-Chlorooctadecane: 40% Spores) imparted significant differences in the mortality of date palm dust mites against different concentrations (*F* = 1345.03; *df* = 4, 48; *p* < 0.0001), time intervals (*F* = 365.97; *df* = 2, 48; < 0.0001), and their interaction (*F* = 77.38; *df* = 8, 48; < 0.0001) as shown in Figure 1III. On the other hand, joint toxicity recorded as a result of Scheme III combinations exhibited bit higher value (joint toxicity = 289) as shown in Table 2. Similarly, strong synergistic interaction (joint toxicity = 713) was recorded from the Scheme IV bioassays in which different concentrations were prepared to finalize the final concentrations with combination strength of 80% 1-Chlorooctadecane: 20% Spores (Table 2). The mortality response of date palm dust mites exposed to different concentrations of these combinations (*F* = 1275.55; *df* = 4, 48; *p* < 0.0001), recorded at different time intervals (*F* = 394.27; *df* = 2, 48; < 0.0001), and their interaction (*F* = 112.46; *df* = 8, 48; < 0.0001), revealed significant differences (Figure 1IV).

### 2.3. Host Antioxidant Defense Response

The exposure of different treatments sole or in different combination schemes produced various levels of catalase (CAT), and superoxide dismutase (SOD) activities relative to control treatment. The relative CAT activities of *O. afrasiaticus* analyzed after 24 h post-exposure revealed significant differences among different treatments (*F* = 256.60; *df* = 5, 120; *p* < 0.0001), concentrations (*F* = 228.63; *df* = 4, 120; *p* < 0.0001), and their interaction (*F* = 2.35; *df* = 20, 120; *p* = 0.0031). Overall, the most potent treatment especially the treatments from the higher concentrations of Scheme IV (80% 1-Chlorooctadecane: 20% Spores) tremendously induced the enzymatic activities of CAT, and remained statistically at the highest level. On the other hand, the lowest concentration of 1-Chlorooctadecane (0.8 mg/mL) after 24 h of post-exposure induced the lowest CAT activities among date palm dust mites (Table 3). In contrast, 0.16 mg/mL of 1-Chlorooctadecane + 3.2 mg/mL of spores induced the lowest CAT activities among date palm dust mites exposed for 72 h. However, all the treatments (*F* = 208.53; *df* = 5, 120; *p* < 0.0001), with different concentrations (*F* = 464.81; *df* = 4, 120; *p* < 0.0001), and their interaction (*F* = 103.21; *df* = 20, 120; *p* < 0.0001), revealed significant differences in the relative CAT activities from 72 h post-exposure date palm dust mites (Table 3). In addition, similar to 24 h post-exposure, date palm dust mites exposed for 72 h also tremendously induced CAT activities in response to the most potent treatment (3.20 mg/mL 1-Chlorooctadecane + 4.0 mg/mL spores). Unlike to relative CAT activities recorded 72 h post-exposure, the most potent treatments including the highest concentrations of Scheme IV (80% 1-Chlorooctadecane: 20% Spores) failed to induce the enzymatic activities of CAT after 120 h post-exposure, and remained statistically at the lowest level (Table 3). At this time interval, *M. anisopliae* EBCL 02049 spores at a concentration of 8 mg/mL induced the highest level of relative CAT activities of infected date palm dust mites, and remained statistically at the highest level. Overall, relative CAT activities recorded after 120 h showed significant differences against different treatments (*F* = 3621.36; *df* = 5, 120; *p* < 0.0001), concentrations (*F* = 1536.63; *df* = 4, 120; *p* < 0.0001), and their interaction (*F* = 105.06; *df* = 20, 120; *p* < 0.0001).

Relative enzymatic activities of SOD recorded from date palm dust mites after 24 h of exposure against different treatments (*F* = 331.28; *df* = 5, 120; *p* < 0.0001), concentrations (*F* = 398.14; *df* = 4, 120; *p* < 0.0001), and their interaction (*F* = 5.44; *df* = 20, 120; *p* < 0.0001), revealed significant differences (Table 4). The infection of the least potent treatment (4 mg/mL spores of *M. anisopliae* EBCL02049) failed to induce SOD activities and remained at the lowest level. On the other hand, 3.20 mg/mL 1-Chlorooctadecane + 4.0 mg/mL spores triggered the SOD activities. This trend continues to increase until 72 h post-exposure, and resulted the highest relative SOD activities from this potent treatment. Furthermore, significant differences in relative SOD activities calculated against different treatments (*F* = 340.46; *df* = 5, 120; *p* < 0.0001), concentrations (*F* = 759.10; *df* = 4, 120; *p* < 0.0001), and their interaction (*F* = 164.03; *df* = 20, 120; *p* < 0.0001), were recorded in this study. 

Overall, the synergistic interaction combinations at their highest concentrations comparatively showed higher relative SOD activities compared with antagonistic interaction combinations (Table 4). On the contrary, relative SOD activities tend to decline after 120 h post-exposure among synergistic interactions compared with antagonistic interaction (Table 4). However, date palm dust mites showed significant differences among relative SOD activities in response to different treatments (*F* = 2230.32; *df* = 5, 120; *p* < 0.0001), concentrations (*F* = 1658.66; *df* = 4, 120; *p* < 0.0001), and their interaction (*F* = 71.62; *df* = 20, 120; *p* < 0.0001).

## 3. Discussion

Plant secondary metabolite 1-Chlorooctadecane improved the efficiency of *M. anisopliae* EBCL 02049 against date palm dust mites by reducing the killing time and increasing killing capacity. However, the extent of pathogenicity that was calculated through joint toxicity varied with the combining proportion of spores with 1-Chlorooctadecane. The simultaneous pairing of *M. anisopliae* EBCL 02049 spores with 1-Chlorooctadecane in a compatible manner as depicted from the biological index in the current study is ideally an interesting alternative approach against such pests, which are difficult to manage with a sole application of fungal spores.

The mortality data of the date palm dust mites in the present study indicated that the sole application of *M. anisopliae* EBCL 02049 produced relatively slow mortality and failed to kill all the tested populations. Slow mortality response of date palm dust mites is in line with what was previously reported against this strain of *M. anisopliae* EBCL 02049 [16]. These findings were further strengthened from previous studies on the virulence of entomopathogenic fungi against date palm dust mites [23,24]. The results of these studies revealed 100% mortality after 15-days post-exposure with *M anisopliae*. Such a long time to impart mortality by the entomopathogenic fungi reported previously [16,24,25], and shown in the current study in the form of low mortality even at the highest concentration (20 mg spores/mL) is demanding an alternative approach to overcome the difficulties in the way of eco-friendly pest management tactics.

Generally, a mixture of plant secondary metabolite with mycopathogen provides eco-friendly stable pest management system compared to environment-deteriorating chemical pesticides. However, the first step to develop a compatible mixture mainly depends on the interaction of these products that might lead to compatible or toxic interaction. The in vitro tests to evaluate the interaction of *M. anisopliae* EBCL 02049 spores with 1-Chlorooctadecane showed encouraging results in laboratory assays. Different concentrations of 1-Chlorooctadecane had little or no effect on the vegetative growth, germination, and sporulation of *M. anisopliae* EBCL 02049. These minor impacts of 1-Chlorooctadecane on growth parameters, which are mainly responsible for the propagation of fungus [26], lead to the value of the biological index under the range of compatible interaction. These compatible interactions at all tested doses of 1-Chlorooctadecane enabled us to support the choice for their simultaneous use in different proportions against date palm dust mites. The search on such a pairing remained a novel option against numerous pest species in order to circumvent slow and low mortality criticism of mycopathogen [23,26,27,28,29,30].

It is well-known that both the fungi and plant secondary metabolites demonstrated an entirely different mode of actions to overcome the target host defense mechanisms to cause mortality. Fungal infection evades the host defense mechanism by triggering complex biochemical interactions in the form of series of events through cuticle adhesion, penetration, proliferation, and toxin production, which ultimately lead to the host mortality [29,31,32,33]. On the other hand, plant secondary metabolites are known antagonists that act by interfering with the signaling of the nervous and cellular systems to overcome the target host defense mechanism [34,35,36,37,38]. Therefore, the combined application is considered as a promising approach due to the differences in their mechanism of actions. Consequently, the mixture of fungal spores and toxin in a compatible manner were evaluated to develop synergistic interaction against various pest species [23,26,28,39,40]. The mixture of *M. anisopliae* EBCL 02049 and 1-Chlorooctadecane evaluated in the current study showed substantial synergistic interaction, which varies with their proportions. The cumulative corrected mortality of date palm dust mites infected with all the mixtures except 0.64 mg/mL 1-Chlorooctadecane + 0.8 mg/mL spores enlisted in Scheme IV (80% 1-Chlorooctadecane: 20% Spores) was more than 95%. Furthermore, LC_50_ results (1.47 mg/mL) upon exposure with the range of mixtures from Scheme IV showed that these combinations more quickly killed the date palm dust mites compared to *M. anisopliae* EBCL 02049 spores and 1-Chlorooctadecane alone. These findings are in line with the previous study on the synergistic interaction of *Beauveria bassiana* spores with Phytol [23]. However, current results are more promising compared to a previous study (Joint toxicity = 691), due to comparatively high toxicity (joint toxicity = 713). Furthermore, the findings of Zou et al., [40] on the joint action of fungal spores and toxin also strengthened our findings, which demonstrated that the synergistic interaction led to a quick mortality response of the combined treatment compared to their sole application. Similarly, the combined treatment enhanced the treatment effect revealed by Hernández et al., [28] against *Tetranychus urticae* further strengthened our findings. Their findings suggested that the compatibility of azadiractin with fungal spores against *Tetranychus urticae* revealed an additive effect, which contributes to improving their control by enhancing the treatment effect in the form of very high mortality. The outcome as a result of this study in the form of synergistic interaction undoubtedly will be useful for targeted management of date palm dust mites using natural products.

The application of the mixture of *M. anisopliae* EBCL 02049 spores and 1-Chlorooctadecane gave a varying antagonistic interaction especially from the bioassays of Scheme I (20% 1-Chlorooctadecane: 80% Spores). There might be a number of explanations for this interaction. Akbar et al., [41], illustrated in their findings that putative antifungal activity of the toxin might contribute towards antagonistic interaction. The studies conducted against *T. urticae* population to find a compatible interaction of fungal spores and toxin revealed that adjustment of toxin concentration is very important to avoid antagonistic interaction [28]. These findings are in line with our results and enabled us to suggest that treatment efficacy should be regulated through optimization of pest management products to develop pest management synergistic interaction.

The antioxidant defense mechanism of the target host is promptly activated to remove the reactive oxygen species produced during stressful situations. The response of antioxidant enzymes calculated from the activities of SOD, and CAT, have shown treatment-specific and time-specific different patterns. The enzymatic activities of the front-line antioxidant enzyme, SOD against oxidative stress revealed distinct patterns at all the tested time intervals. The most potent treatment established in the current study significantly induced SOD activities until 72 h post-exposure. However, the lateral stage nutrient-deficient date palm dust mites failed to induce relative SOD activities and remained significantly at the lowest level. The induction of SOD activities under stressful situations has already been reported from several mite species. For instance, SOD activities in *Tetranychus cinnabarinus* fed on transgenic cassava lines resistant to this mite species were significantly induced compared with control mites [42]. The recent findings on the antioxidant response of date palm dust mites upon exposure with fungal infections with variable virulence range strengthened our findings by revealing similar patterns of SOD activities from potent treatments as depicted in the current study [24]. The initial increase in SOD activities established here and elsewhere mainly aimed to minimize the cellular damage by regulating the traffic of ROS generated as a result of oxidative stress by converting ROS into H_2_O_2_. In the meanwhile, CAT is activated as described too in the current study, which detoxifies hydrogen peroxide by transforming into oxygen and water [43]. The least potent treatment in this study revealed high CAT activities over time for the safe removal of ROS corroborates with Hussain et al., [16]. On the other hand, enhanced CAT activities from the most potent treatment at the initial and middle staged infection, while negligible CAT activities from the late-staged nutrient-deficient infected mites are in agreement with previous studies conducted on different mite species on the exploration of antioxidant defense mechanism [16,23,24,42]. Such a pattern of defense enzymes may suggest that toxicity of the treatments regulates the host antioxidant enzyme activities.

## 4. Materials and Methods 

### 4.1. Date Palm Dust Mites

Populations of *O. afrasiaticus* for laboratory experimentation were directly collected from NCPD (National Center for Palms & Dates), Al-Ahsa, Kingdom of Saudi Arabia. These populations were kept at 25 ± 1 °C; 62.5 ± 12.5% RH, as mentioned in the previous study [24].

### 4.2. M. anisopliae

The strain EBCL 02049 of *M. anisopliae* isolated from *Coptotermes formosanus* Shiraki during 2002 from Jiangxi, China was grown on potato dextrose agar for twenty-four days under controlled conditions (25 ± 0.5 °C; 70 ± 5% RH). This particular strain was selected because their spores suspension has shown virulence against *Ocinara varians* Walker [13,44], *Coptotermes formosanus* Shiraki [14,15], and *O. afrasiaticus* (McGregor) [16]. The spore suspensions of required concentrations were prepared using 0.05% Tween 80 (Sigma–Aldrich, London, UK) by Neubauer hemocytometer (Wertheim, Germany).

### 4.3. 1-Chlorooctadecane

1-Chlorooctadecane (CH_3_ (CH_2_) 16CH_2_ Cl) was screened from our preliminary study (data not shown) on the chemical fractionation of *Cucumis sativus* that showed toxicity against date palm dust mites (data not shown). The identified fraction, 1-Chlorooctadecane, selected for the current study was purchased from Sigma–Aldrich in pure form (Cat # 238368; CAS Number 3386-33-2) to perform compatibility, toxicity, and synergistic studies. The stock solution of the 1-Chlorooctadecane was prepared by dissolving in ethanol.

### 4.4. Compatibility of M. anisopliae Spores with 1-Chlorooctadecane

The potato dextrose agar plates (90-mm × 15 mm) with different concentrations (0.80, 1.60, 2.40, 3.20, and 4.00 mg/mL) of 1-Chlorooctadecane were prepared as described in detail by a previous study [45] Ten microliter spores suspension of *M. anisopliae* EBCL 02049 at a single concentration (1 × 10^6^ spores/mL) were pipetted in the center of each PDA-plate. The plates after inoculation were sealed with parafilm. All the plates were incubated in complete darkness at 25 ± 0.5 °C; 70 ± 5% RH. The values of three parameters including sporulation (spores/mL), germination (%), and vegetative growth (mm), were recorded in order to calculate biological index (BI) [23]. Ten replicates were prepared likewise separately for each parameter using ten PDA-plates. 

In the case of percent germination, petri plates were incubated for 12 h post-inoculation. Percent germination (GR) of *M. anisopliae* EBCL 02049 spores was calculated by counting a hundred spores under seven different fields of vision. On the other hand, vegetative growth (VG) and sporulation (SP) was determined after 12-days post-inoculation. Seven perpendicular radial growths (mm) of each fungal culture were measured with the help of a transparent ruler for vegetative growth (VG) determination. Furthermore, a 15 mm diameter sporulating culture of *M. anisopliae* EBCL 02049 was taken using 15 mm sterilized cork borer in order to calculate sporulation with the help of Neubauer hemocytometer using 0.05% Tween 80 solution, under a compound microscope. All the parameters including sporulation, percent germination, and vegetative growth were separately analyzed by One-way Analysis of Variance, and their means by Fisher’s LSD test (α = 0.05). 

The BI was calculated by adding the values of above-mentioned parameters into the standard formula (BI = [47 × VG + 43 × SP + 10 × GR]/100). As per standard criterion, BI > 66 ranked as Compatible interaction, while BI < 42 ranked as a toxic interaction. The BI between 42 to 66 is ranked as Moderately Toxic interaction [46].

### 4.5. Laboratory Evaluation of M. anisopliae Spores with 1-Chlorooctadecane in Different Proportions against Date Palm Dust Mites

Concentration–mortality response bioassays were performed in order to find the most potent fungal–toxin interaction. The treatments with different proportions used for leaf-dip bioassays are mentioned in Table 5. Each treatment (50 mL) sole or in different combinations mentioned in Scheme I, II, III, and IV was prepared separately in a sterilized glass beaker. Pesticide-free leaf-discs of date palm (7.5 cm length × 4 cm width) were dipped with the help of a forceps. After air drying, fifty deutonymphs (second nymphal stage) date palm dust mites were transferred with the help of a camel hair brush on these exposed leaf-discs surrounded with wet cotton in a petri dish (150 mm × 20 mm). Control leaf-discs were dipped in their respective solvent. In case of sole application of spores, 0.05% tween 80 was used as control treatment, while control treatment for 1-Chlorooctadecane was prepared using ethanol with strength used to prepare stock solution of 1-Chlorooctdecane. Control treatment for mixtures was separately prepared using Tween 80 and ethanol with their respective strengths to dissolve them. All the experimental units were maintained at 28.0 ± 0.5 °C with a photoperiod of 16:8 h (L:D). Five replicates were prepared likewise. Mortality data were recorded daily for 10 days post-exposure. Dead mites were transferred into the petri dishes lined with dampened sterile filter paper. Mycosis of the inoculating fungal isolate was confirmed by microscopic examination of the corpses. The Abbott formula was applied in order to correct the treatment mortality data from their respective control mortality data [47]. While each treatment mortality data were used to calculate LC_50_ (Lethal Concentration to impart 50% mortality of date palm dust mites) by Probit analysis [48]. Furthermore, mortality data after angular transformation were analyzed by Repeated Measures ANOVA, and means by Fisher’s LSD test [49]. Interaction of different proportions with multiple concentrations as mentioned in Table 5 was designed mainly to calculate joint toxicity as described by Sun et al. [50]. 

### 4.6. Exploration of Host Antioxidant Defense Response

Second nymphal stage date palm dust mites infected separately with different treatments mentioned in Table 5 were allowed to feed on date palm leaf-discs at 28.0 ± 0.5 °C with a photoperiod of 16:8 h (L:D). Five replicates were prepared. Samples of live date palm dust mites were taken after 24 h, 72 h, and 120 h in order to calculate CAT and SOD activities. Each sample was individually homogenized in ice-cold potassium phosphate buffer saline. The tissues were crushed by a glass homogenizer. All the samples were centrifuged (12,000× *g*) for 15 min. The supernatants were served as enzyme source and taken for protein quantification by a standard method [51]. The enzymatic responses of CAT (Cat # CAT100-1KT, Sigma–Aldrich, London, UK), and SOD (Cat # 19160-1KT-F, Sigma–Aldrich, London, UK), upon exposure with different treatments were calculated using the standard protocols provided by the manufacturer of above-mentioned kits. The enzymatic activities in each treatment were expressed in percentages, which are relative to the enzymatic activities data obtained from their respective control treatments. Two-factor factorial analysis was used to analyze each treatment relative to control treatment enzymatic activities of date palm dust mites, and their significant differences among different treatments by Fisher’s LSD test [52].

## 5. Conclusions

In conclusion, bioassay results of the present study demonstrated that *M. anisopliae* EBCL 02049 was found to be compatible with all tested concentrations of 1-Chlorooctadecane by showing a very high value of biological index (BI > 82). Our results demonstrated that combined application of *M. anisopliae* spores with 1-Chlorooctadecane resulted in enhanced treatment efficacy against second nymphal stage of date palm dust mites. However, we found that higher proportion of 1-Chlorooctadecane (80%) and lower proportion of *M. anisopliae* spores (20%) greatly improved the synergism (joint toxicity = 713). Furthermore, their synergism led to the most potent interaction, which reduced the time and concentration to cause high mortality among date palm dust mites by altering their antioxidant defense mechanism. Hence, the joint action of *M. anisopliae* spores and 1-Chlorooctadecae could be a promising component of integrated management of date palm dust mites.

## Figures and Tables

**Table 1:** Compatibility of 1-Chlorooctadecane with the spores of *M. anisopliae*.

Treatments	Vegetative Growth (mm) ^1^	Germination (%) ^1^	Sporulation (×10^6^ Spores/mL) ^1^	Biological Index	Classification ^2^
Control	85.70 ± 2.25^a^	98.70 ± 0.79^a^	7.80 ± 0.63^a^	-	-
0.8 mg/mL	84.40 ± 2.28^ab^	98.20 ± 0.99^a^	7.30 ± 0.60^ab^	96.48	Compatible
1.6 mg/mL	84.20 ± 21.69^ab^	97.90 ± 1.03^a^	6.40 ± 0.54^abc^	91.38	Compatible
2.4 mg/mL	79.30 ± 1.82^bc^	97.30 ± 0.71^a^	6.20 ± 0.61^bc^	87.53	Compatible
3.2 mg/mL	78.10 ± 2.11^c^	96.70 ± 0.93^a^	5.60 ± 0.40^c^	83.50	Compatible
4.0 mg/mL	77.70 ± 1.54^c^	97.20 ± 0.81^a^	5.50 ± 0.34^c^	82.78	Compatible

^1^ Numerical values (means ± SE) are the means of ten replicates. Different lower-case letter(s) superscript followed by means ± SE within a column are significantly different (Fisher’s LSD test; α = 0.05). ^2^ Biological index-based classification criterion: BI more than 66 classified as Compatible, BI between 42 to 66 classified as Moderately toxic; BI less than or equal to 42 classified as Toxic interaction.

**Table 2:** Toxicity of the interactions between 1-Chlorooctadecane and the spores of *M. anisopliae* against date palm dust mites.

Combinations	LC_50_ (mg/mL)	Joint Toxicity	Interaction ^1^
**Scheme I: 20% 1-Chlorooctadecane: 80% Spores**			
0.16 mg/mL 1-Chlorooctadecane + 3.2 mg/mL spores	8.75 (7.10–10.75)	47	Antagonistic
0.32 mg/mL 1-Chlorooctadecane + 6.4 mg/mL spores			
0.48 mg/mL 1-Chlorooctadecane + 9.6 mg/mL spores			
0.64 mg/mL 1-Chlorooctadecane + 12.8 mg/mL spores			
0.80 mg/mL 1-Chlorooctadecane + 16.0 mg/mL spores			
**Scheme II: 40% 1-Chlorooctadecane: 60% Spores**			
0.32 mg/mL 1-Chlorooctadecane + 2.4 mg/mL spores	4.62 (3.41–5.65)	112	Synergistic
0.64 mg/mL 1-Chlorooctadecane + 4.8 mg/mL spores			
0.96 mg/mL 1-Chlorooctadecane + 7.2 mg/mL spores			
1.28 mg/mL 1-Chlorooctadecane + 9.6 mg/mL spores			
1.60 mg/mL 1-Chlorooctadecane + 12 mg/mL spores			
**Scheme III: 60% 1-Chlorooctadecane: 40% Spores**			
0.48 mg/mL 1-Chlorooctadecane + 1.6 mg/mL spores	2.39 (2.00–2.72)	289	Synergistic
0.96 mg/mL 1-Chlorooctadecane + 3.2 mg/mL spores			
1.44 mg/mL 1-Chlorooctadecane + 4.8 mg/mL spores			
1.92 mg/mL 1-Chlorooctadecane + 6.4 mg/mL spores			
2.40 mg/mL 1-Chlorooctadecane + 8.0 mg/mL spores			
**Scheme IV: 80% 1-Chlorooctadecane: 20% Spores**			
0.64 mg/mL 1-Chlorooctadecane + 0.8 mg/mL spores	1.47 (1.24–1.67)	713	Synergistic
1.28 mg/mL 1-Chlorooctadecane + 1.6 mg/mL spores			
1.92 mg/mL 1-Chlorooctadecane + 2.4 mg/mL spores			
2.56 mg/mL 1-Chlorooctadecane + 3.2 mg/mL spores			
3.20 mg/mL 1-Chlorooctadecane + 4.0 mg/mL spores			

^1^ Interaction criterion: Joint toxicity less than 100 is classified as Antagonistic.

**Table 3:** Relative catalase (CAT) activities of date palm dust mites fed for different time intervals on date palm leaf-discs treated solely and in different combinations of 1-Chlorooctadecane with *M. anisopliae* spores.

Treatments	Post-Exposure Duration		
	24 h ^1^	72 h ^1^	120 h ^1^
**1-Chlorooctadecane**	(%)	(%)	(%)
0.8 mg/mL	13.35 ± 0.55^p^	32.18 ± 0.34^m^	46.03 ± 0.80^g^
1.6 mg/mL	20.32 ± 1.02^lm^	35.56 ± 0.49^l^	39.59 ± 0.88^i^
2.4 mg/mL	21.17 ± 1.03^kl^	40.09 ± 0.69^ij^	23.27 ± 0.44^l^
3.2 mg/mL	24.38 ± 1.18^hi^	46.16 ± 0.97^g^	17.13 ± 0.34^op^
4.0 mg/mL	27.14 ± 1.32^fg^	52.86 ± 0.92^d^	15.14 ± 0.25^q^
**Scheme I: 20% 1-Chlorooctadecane: 80% Spores**			
0.16 mg/mL 1-Chlorooctadecane + 3.2 mg/mL spores	15.04 ± 0.21^op^	31.52 ± 0.37^m^	60.78 ± 0.94^b^
0.32 mg/mL 1-Chlorooctadecane + 6.4 mg/mL spores	16.38 ± 0.24^no^	39.64 ± 0.59^j^	53.88 ± 0.98^e^
0.48 mg/mL 1-Chlorooctadecane + 9.6 mg/mL spores	20.22 ± 0.54^lm^	44.17 ± 0.72^gh^	56.42 ± 0.93^d^
0.64 mg/mL 1-Chlorooctadecane + 12.8 mg/mL spores	21.19 ± 0.78^kl^	50.11 ± 0.95^ef^	52.78 ± 0.95^e^
0.80 mg/mL 1-Chlorooctadecane + 16.0 mg/mL spores	25.15 ± 0.92^ghi^	43.11 ± 0.84^h^	41.46 ± 0.76^h^
**Scheme II: 40% 1-Chlorooctadecane: 60% Spores**			
0.32 mg/mL 1-Chlorooctadecane + 2.4 mg/mL spores	20.32 ± 0.31^lm^	35.30 ± 0.55^l^	40.03 ± 0.64^hi^
0.64 mg/mL 1-Chlorooctadecane + 4.8 mg/mL spores	22.06 ± 0.70^jkl^	36.50 ± 0.57^l^	29.74 ± 0.55^j^
0.96 mg/mL 1-Chlorooctadecane + 7.2 mg/mL spores	27.15 ± 0.72^fg^	39.68 ± 0.78^j^	24.09 ± 0.50^l^
1.28 mg/mL 1-Chlorooctadecane + 9.6 mg/mL spores	30.01 ± 0.90^de^	48.45 ± 0.91^f^	20.10 ± 0.24^m^
1.60 mg/mL 1-Chlorooctadecane + 12 mg/mL spores	34.18 ± 0.92^bc^	52.93 ± 0.93^d^	15.20 ± 0.20^q^
**Scheme III: 60% 1-Chlorooctadecane: 40% Spores**			
0.48 mg/mL 1-Chlorooctadecane + 1.6 mg/mL spores	20.10 ± 0.24^lm^	35.69 ± 0.46^l^	26.54 ± 0.72^k^
0.96 mg/mL 1-Chlorooctadecane + 3.2 mg/mL spores	24.19 ± 0.60^hij^	37.43 ± 0.56^kl^	24.19 ± 0.60^l^
1.44 mg/mL 1-Chlorooctadecane + 4.8 mg/mL spores	29.12 ± 0.78^ef^	42.02 ± 0.70^hi^	20.29 ± 0.39^m^
1.92 mg/mL 1-Chlorooctadecane + 6.4 mg/mL spores	32.22 ± 0.87^cd^	55.90 ± 0.76^c^	17.44 ± 0.32^no^
2.40 mg/mL 1-Chlorooctadecane + 8.0 mg/mL spores	33.22 ± 0.90^bc^	63.85 ± 0.93^b^	09.79 ± 0.26^r^
**Scheme IV: 80% 1-Chlorooctadecane: 20% Spores**			
0.64 mg/mL 1-Chlorooctadecane + 0.8 mg/mL spores	23.08 ± 0.57^ijk^	38.92 ± 0.57^jk^	15.58 ± 0.28^pq^
1.28 mg/mL 1-Chlorooctadecane + 1.6 mg/mL spores	29.37 ± 0.64^ef^	43.35 ± 0.69^h^	09.67 ± 0.25^r^
1.92 mg/mL 1-Chlorooctadecane + 2.4 mg/mL spores	33.02 ± 0.81^bc^	50.16 ± 0.79^ef^	03.58 ± 0.24^s^
2.56 mg/mL 1-Chlorooctadecane + 3.2 mg/mL spores	35.12 ± 0.93^b^	63.96 ± 0.91^b^	01.79 ± 0.15^t^
3.20 mg/mL 1-Chlorooctadecane + 4.0 mg/mL spores	39.32 ± 1.22^a^	71.64 ± 1.05^a^	01.24 ± 0.13^t^
***M. anisopliae* EBCL02049 spores**			
4 mg/mL	15.34 ± 0.43^op^	49.13 ± 0.81^f^	58.19 ± 0.81^c^
8 mg/mL	18.27 ± 0.56^mn^	57.16 ± 0.98^c^	63.83 ± 0.77^a^
12 mg/mL	21.08 ± 0.78^kl^	51.35 ± 0.97^de^	48.38 ± 0.97^f^
16 mg/mL	23.26 ± 0.88^ijk^	43.67 ± 0.93^h^	28.07 ± 0.62^jk^
20 mg/mL	26.17 ± 0.91^gh^	36.26 ± 0.68^l^	19.13 ± 0.60^mn^

^1^ Numerical values (means ± SE) are the means of five replicates. Different lower-case letter(s) superscript followed by means ± SE of relative CAT activities of *O. afrasiaticus* within the column are significantly different (Fisher’s LSD test; α = 0.05).

**Table 4:** Relative superoxide dismutase (SOD) activities of date palm dust mites fed for different time intervals on date palm leaf-discs treated solely and in different combinations of 1-Chlorooctadecane with *M. anisopliae* spores.

Treatments	Post-Exposure Duration		
	24 h ^1^	72 h ^1^	120 h ^1^
**1-Chlorooctadecane**	(%)	(%)	(%)
0.8 mg/mL	12.89 ± 0.53^kl^	30.38 ± 0.85^q^	66.27 ± 1.19^bc^
1.6 mg/mL	20.42 ± 0.78^h^	36.15 ± 0.90^op^	57.13 ± 1.21^d^
2.4 mg/mL	21.20 ± 0.87^h^	43.85 ± 0.93^l^	37.31 ± 0.65^h^
3.2 mg/mL	26.87 ± 1.05^d^	54.04 ± 1.27^g^	31.36 ± 0.45^ij^
4.0 mg/mL	29.65 ± 1.29^c^	66.10 ± 1.35^c^	19.77 ± 0.36^lm^
**Scheme I: 20% 1-Chlorooctadecane: 80% Spores**			
0.16 mg/mL 1-Chlorooctadecane + 3.2 mg/mL spores	11.17 ± 0.27^l^	53.13 ± 0.97^gh^	76.13 ± 1.01^a^
0.32 mg/mL 1-Chlorooctadecane + 6.4 mg/mL spores	13.33 ± 0.42^k^	61.18 ± 0.99^e^	68.07 ± 0.98^b^
0.48 mg/mL 1-Chlorooctadecane + 9.6 mg/mL spores	18.13 ± 0.50^j^	52.54 ± 1.04^gh^	57.30 ± 1.11^d^
0.64 mg/mL 1-Chlorooctadecane + 12.8 mg/mL spores	20.11 ± 0.76^hi^	46.57 ± 0.95^j^	40.43 ± 0.69^g^
0.80 mg/mL 1-Chlorooctadecane + 16.0 mg/mL spores	21.32 ± 0.85^gh^	29.46 ± 0.50^q^	25.30 ± 0.58^k^
**Scheme II: 40% 1-Chlorooctadecane: 60% Spores**			
0.32 mg/mL 1-Chlorooctadecane + 2.4 mg/mL spores	14.17 ± 0.61^k^	37.27 ± 0.61^o^	43.08 ± 0.82^f^
0.64 mg/mL 1-Chlorooctadecane + 4.8 mg/mL spores	21.20 ± 0.63^h^	49.65 ± 0.81^i^	44.12 ± 0.96^f^
0.96 mg/mL 1-Chlorooctadecane + 7.2 mg/mL spores	25.42 ± 0.91^de^	58.40 ± 1.18^f^	39.80 ± 0.88^g^
1.28 mg/mL 1-Chlorooctadecane + 9.6 mg/mL spores	27.07 ± 0.93^d^	61.44 ± 1.20^e^	25.46 ± 0.51^k^
1.60 mg/mL 1-Chlorooctadecane + 12.0 mg/mL spores	30.11 ± 1.02c	64.55 ± 1.25^cd^	21.42 ± 0.50^l^
**Scheme III: 60% 1-Chlorooctadecane: 40% Spores**			
0.48 mg/mL 1-Chlorooctadecane + 1.6 mg/mL spores	17.21 ± 0.39^j^	41.14 ± 0.77^mn^	36.26 ± 0.68^h^
0.96 mg/mL 1-Chlorooctadecane + 3.2 mg/mL spores	23.10 ± 0.65^fg^	44.39 ± 0.85^kl^	29.32 ± 0.63^j^
1.44 mg/mL 1-Chlorooctadecane + 4.8 mg/mL spores	24.27 ± 0.70^ef^	54.25 ± 1.03^g^	25.37 ± 0.53^k^
1.92 mg/mL 1-Chlorooctadecane + 6.4 mg/mL spores	29.22 ± 0.95^c^	63.76 ± 1.24^d^	20.54 ± 0.43^l^
2.40 mg/mL 1-Chlorooctadecane + 8.0 mg/mL spores	34.20 ± 1.02^a^	72.26 ± 1.25^b^	16.28 ± 0.25^n^
**Scheme IV: 80% 1-Chlorooctadecane: 20% Spores**			
0.64 mg/mL 1-Chlorooctadecane + 0.8 mg/mL spores	23.38 ± 0.52^f^	43.15 ± 0.81^lm^	32.41 ± 0.52^i^
1.28 mg/mL 1-Chlorooctadecane + 1.6 mg/mL spores	25.32 ± 0.70^de^	53.19 ± 0.91^gh^	26.42 ± 0.43^k^
1.92 mg/mL 1-Chlorooctadecane + 2.4 mg/mL spores	26.19 ± 0.98^d^	60.45 ± 0.99^ef^	21.24 ± 0.37^l^
2.56 mg/mL 1-Chlorooctadecane + 3.2 mg/mL spores	32.34 ± 1.12^b^	66.23 ± 1.10^c^	18.03 ± 0.52^mn^
3.20 mg/mL 1-Chlorooctadecane + 4.0 mg/mL spores	35.41 ± 1.18^a^	81.28 ± 1.26^a^	11.80 ± 0.37^o^
***M. anisopliae* EBCL02049 spores**			
4 mg/mL	09.28 ± 0.34^m^	34.20 ± 0.79^p^	56.53 ± 0.93^d^
8 mg/mL	17.11 ± 0.48^j^	40.14 ± 0.88^n^	65.87 ± 1.03^c^
12 mg/mL	18.41 ± 0.58^ij^	46.43 ± 0.92^jk^	54.14 ± 0.97^e^
16 mg/mL	20.13 ± 0.66^hi^	51.24 ± 0.98^hi^	43.75 ± 0.99^f^
20 mg/mL	23.16 ± 0.89^fg^	60.24 ± 1.14^ef^	25.46 ± 0.72^k^

^1^ Numerical values (means ± SE) are the means of five replicates. Different lower-case letter(s) superscript followed by means ± SE of relative SOD activities of *O. afrasiaticus* within the column are significantly different (Fisher’s LSD test; α = 0.05).

**Table 5:** Various bioassay schemes evaluated against date palm dust mites.

1-Chlorooctadecane (mg/mL)	Interaction Schemes								*M. anisopliae* (mg/mL)
	Scheme I		Scheme II		Scheme III		Scheme IV		
	1-Chlorooctadecane (20%): Spores (80%)		1-Chlorooctadecane (40%): Spores (60%)		1-Chlorooctadecane (60%): Spores (40%)		1-Chlorooctadecane (80%): Spores (20%)		
	Toxin mg/mL	Spores mg/mL	Toxin mg/mL	Spores mg/mL	Toxin mg/mL	Spores mg/mL	Toxin mg/mL	Spores mg/mL	
0.8	0.16	3.2	0.32	2.4	0.48	1.6	0.64	0.8	4
1.6	0.32	6.4	0.64	4.8	0.96	3.2	1.28	1.6	8
2.4	0.48	9.6	0.96	7.2	1.44	4.8	1.92	2.4	12
3.2	0.64	12.8	1.28	9.6	1.92	6.4	2.56	3.2	16
4	0.8	16	1.6	12	2.4	8	3.2	4	20
